# Biological markets in cooperative breeders: quantifying outside options

**DOI:** 10.1098/rspb.2017.0904

**Published:** 2017-06-14

**Authors:** Lena Grinsted, Jeremy Field

**Affiliations:** School of Life Sciences, University of Sussex, John Maynard Smith Building, Falmer, Brighton BN1 9QG, UK

**Keywords:** social insects, economics, partner choice, competition, group living, trade

## Abstract

A major aim in evolutionary biology is to understand altruistic help and reproductive partitioning in cooperative societies, where subordinate helpers forego reproduction to rear dominant breeders' offspring. Traditional models of cooperation in these societies typically make a key assumption: that the only alternative to staying and helping is solitary breeding, an often unfeasible task. Using large-scale field experiments on paper wasps (*Polistes dominula*), we show that individuals have high-quality alternative nesting options available that offer fitness payoffs just as high as their actual chosen options, far exceeding payoffs from solitary breeding. Furthermore, joiners could not easily be replaced if they were removed experimentally, suggesting that it may be costly for dominants to reject them. Our results have implications for expected payoff distributions for cooperating individuals, and suggest that biological market theory, which incorporates partner choice and competition for partners, is necessary to understand helping behaviour in societies like that of *P. dominula*. Traditional models are likely to overestimate the incentive to stay and help, and therefore the amount of help provided, and may underestimate the size of reproductive concession required to retain subordinates. These findings are relevant for a wide range of cooperative breeders where there is dispersal between social groups.

## 1. Introduction

Altruistic helping behaviour occurs throughout the animal kingdom despite costs to helpers' direct fitness. In cooperatively breeding animals, subordinates care for, defend and provision the offspring of dominant breeders, while foregoing or delaying their own reproduction [1,2]. A range of factors has been identified to explain the evolution and maintenance of this phenomenon, including both direct fitness benefits, such as inheritance of the breeding position [3,4], and indirect fitness benefits obtained through helping a relative [5,6]. However, there is an increasing awareness in the literature of the limitations of traditional theoretical models, and a call for more complex models that more realistically describe the social environment of individuals [7–12]. Specifically, traditional models predicting the level of help and reproductive skew in cooperative breeders often make a key assumption: that a subordinate helper's only alternative to staying and helping in its current group is to leave and breed solitarily [13–18]. However, breeding alone is often unfeasible or highly risky [3,19,20], leading to the prediction that subordinates should accept a high workload and a small share or zero part of the reproduction, in order to remain in the group.

Recent literature increasingly suggests that in order to correctly estimate the costs and benefits associated with staying and helping in a group, one must compare the payoffs of that decision with an individual's true alternative options [7,8,11,21]. In reality, a subordinate's alternative options may include switching to another group or recruiting other cooperative partners to initiate a new breeding group [11,20,22,23]. If such alternative options could lead to higher fitness payoffs than solitary breeding, payoff distributions may have been miscalculated in past studies, overestimating the incentive for subordinates to stay and help. Hence, future studies are encouraged to include the following: partner choice rather than partner control, where sanctioning of uncooperative partners is replaced by partner switching [12,23–25]; outside options beyond solitary breeding [9,10]; asymmetric relationships where the exchange of behaviours is more valuable for one of the parties [21,26,27]; and *n*-player interactions not achievable in traditional 2-player cooperative games [21,28]. These modifications can be achieved by invoking biological market models [21]. Biological market theory predicts that competition for cooperative partners will affect the value of commodities exchanged between individuals of different trader classes [9,10]. In cooperative breeders, subordinates may be seen as effectively exchanging helping behaviour for group membership [15,29,30], and the value of helping behaviour may therefore be affected by the supply of and demand for help in the market. Subordinates may be described as ‘paying to stay’ [30–32] or dominants as ‘paying for help’ [33,34], depending on which commodity is in focus. For example, when there is competition among dominants for a limited supply of helpers so that help is in high demand, dominants may be willing to accept subordinates paying less for group membership through reducing their work efforts. Similarly, dominants might be willing to pay more for help by granting a higher share of reproduction to subordinates [9,10,22,33,34].

Several studies of cooperatively breeding mammals [31,33,34], fishes [32,35,36], birds [30,37,38] and insects [22] have found support for the concept that dominants and subordinates exchange commodities as described above. In the cooperatively breeding paper wasp, *Polistes dominula*, we previously reported data consistent with the hypothesis that dominants have to accept a lower payment from their subordinates when competition for help is increased in the population [22]. We first showed that wasps had outside options and a choice of cooperative partners. We then experimentally increased the amount of outside options available to subordinates and found that subordinates, as a result, decreased their work efforts [22]. These results suggest that there is a biological market in this species where the supply of outside options affects the exchange of cooperative behaviours within groups. However, in order to wholly understand the dynamics between dominants and subordinates, we need to know not only the number of alternative options available but also their quality. Only by evaluating the attractiveness of outside options will the behavioural decisions of cooperative partners be clear. Here, we quantify the outside options available to *P. dominula* wasps and estimate the fitness payoffs associated with these options. We further evaluate how partner choice may affect the payoff distribution between cooperative partners, and assess the implications this may have for cooperative theory.

The nesting behaviour and social organization of *P. dominula* is well studied, and our study sites offer large samples of small groups [8,22,39]. At these sites, thousands of mated females from the same generation emerge simultaneously from hibernation in early spring and found hundreds of nests along cactus hedges (*Opuntia ficus-indica*). Groups of typically fewer than 10 females and small numbers (approx. 6.4% of all females in [40]) of solitary breeders rear workers that mature during late spring and early summer. Here we focus on the pre-worker stage where groups of similar-aged females live as cooperative breeders. The dominant breeder lays all or most of the eggs, while subordinates build and expand the nest, forage and help care for the offspring of the dominant [41]. Nest residents often consist of genetically related individuals (sisters and cousins), but a significant proportion of subordinates are unrelated to the dominant they are helping [42–44]. The chance of inheriting the breeding position or obtaining a small share of the reproduction has been used to explain the presence of unrelated helpers in this species: Leadbeater *et al*. [3] found that the amount of direct fitness obtained as a subordinate was greater than through solitary breeding [3]. However, if helpers have alternative options available that offer higher fitness payoffs than solitary breeding, the incentive to stay and help in their current groups may previously have been overestimated.

We ask the following questions. (i) Do available nesting options include high-payoff alternatives? Alternative options will affect the predictions of existing models only if they offer a higher payoff than solitary nesting. (ii) Do alternative options differ from observed choices in ways that should affect direct and indirect fitness, such as inheritance rank and relatedness to the dominant? We predict that alternative options are inferior to observed choices: in a biological market, individuals are expected to assess their options and make the choice that offers the highest payoff [10]. (iii) Is it costly for dominants to reject an additional cooperative partner? We expect help to be in high demand because productivity and group survival increase with the number of helpers in *P. dominula* [3,22], so we predict that rejecting a joiner represents a cost to dominant breeders.

## 2. Methods

### (a) Study species, field site and handling of animals

*Polistes dominula* is a primitively eusocial (cooperatively-breeding) wasp lacking morphological castes. At our field site, females from the same generation found nests in early spring after overwintering. The first female offspring to mature in late spring become workers and those maturing during summer mate and overwinter, to restart the cycle next spring [3].

**Figure 1.**
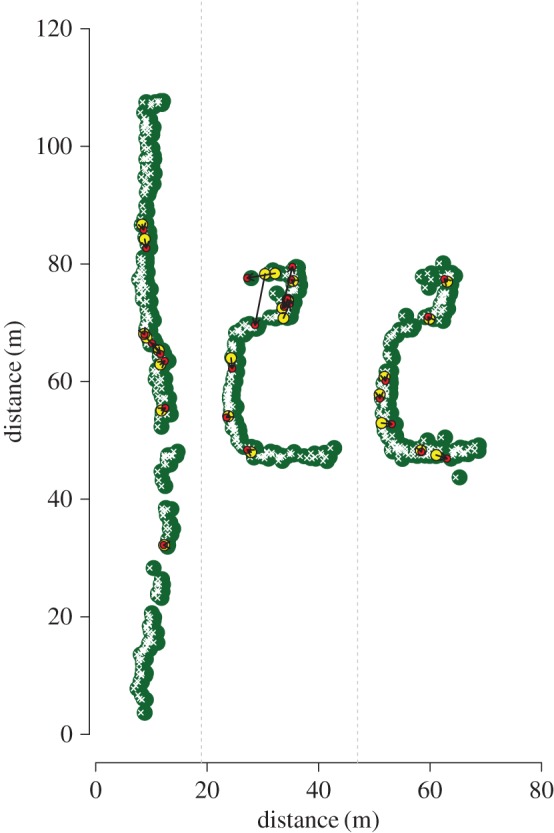
Map of all nests in the three sub-populations used during two different field season in 2013 (left hand and middle sections) and 2014 (right hand section). Cactus hedges are indicated in green and nests as white Xs. Second-choice joiners' first nest choices are indicated in yellow and their second choices in red, with an arrow connecting the two.

Experiments were carried out in a rural area in southern Spain, close to Conil de la Frontera, Cadiz (N 36°17′10.9″ W 6°03′57.8″) [3,22] during two field seasons: March–May 2013 and 2014. We tagged and numbered a total of approximately 700 nests: approximately 475 nests in two sub-populations in 2013 and approximately 225 nests in one subpopulation in 2014 (figure 1; same data as used for ‘the partner choice experiment’ in [22]). We further recorded the location of all nests along three axes (to nearest 5 cm), allowing us to calculate the distances between nests in a 3D space.

Combining the two field seasons, individuals from approximately 200 of these nests were collected during early mornings, before sunrise (6.00–7.00). In the laboratory we gave each wasp a unique code of four coloured dots on her thorax using enamel paints; measured the length of one of her wings to the nearest 0.1 mm; and obtained a DNA sample by cutting the tarsus from a middle leg. Tarsus samples were kept in 100% ethanol at approximately 4°C until used for genotyping. Wasps were released close to their nests the same morning before 11.00. When wasps were permanently removed as a part of an experimental treatment they were either freeze-killed or released at a field site 2.5 km away: none returned to her original site.

### (b) Experimental set-up

The day after residents were marked on a nest, we checked the nest in the evening for additional, unmarked residents. Any unmarked residents were collected the following morning and marked as described above. Once all nest residents were marked, we started daytime and evening censuses. Daytime censuses consisted of 3–4 spot-checks per day (min. 30 min between each census) on sunny days every 2–4 days, where the presence or absence of nest residents were recorded during the main foraging period (11.00–17.00). From the daytime censuses we identified the social rank of each resident in the linear dominance hierarchy: the dominant breeder spends the most time on the nest, while the lowest-ranked individual spends the most time away from the nest foraging [45].

In the evenings, nest residents return to their nests for the night. During evening censuses (18.00–20.00), performed every 2–4 days, we searched focal nests for new joiners. To mark a new joiner with minimal disturbance, we carefully applied a single pink paint dot to its abdomen while it was on the nest (day 0 of the joiner experiment). We videoed a subset of 21 focal nests on day 1 for three hours during the main foraging period (11.00–17.00). The following morning (day 2), we caught and marked the joiner as described above. On day 3 we confirmed the presence of the joiner during an evening census, so that we could plan to apply treatment the following morning (day 4; treatment morning). If the joiner was not present on its nest during one of these checks, we looked for it on the nest for a maximum of three days. If the joiner re-appeared within this period we continued with the next step of the procedure; if it did not we resumed normal censuses of the nest.

On the morning of treatment in the joiner experiment, we applied one of three treatments (*n* treated focal nests = 62). (1) *Joiner's first choice*: this was our control treatment where the joiner was allowed to stay and no nest residents were removed. (2) *Joiner's second choice*: we removed the joiner's first nest choice by permanently removing the nest and all of its residents, while immediately releasing the joiner itself. If any established residents were absent from the nest, we left the nest *in situ* for a maximum of 48 hours before removing it, allowing us to attract and remove remaining residents. (3) *Joiner removal*: we permanently removed the joiner (or both joiners if two had joined) while releasing all other residents near to the nest.

In addition to applying one of the three described treatments, we also recorded the presence of all residents on focal nests by collecting all wasps on their nest, recording their IDs and releasing individuals immediately according to treatment. We further performed a brood census on each focal nest, which included counting the number of cells and categorizing the development of brood within each cell. Nest-level brood values were later summed as follows: small larva (given a value of 1.5), medium larva (2), large larva (3) and pupa (4); a cell without a larva or pupa was assumed to contain an egg (1).

In addition to the joiner experiment, we carried out a subordinate experiment similar to treatment 2 (joiner's second choice), but using established low-ranking subordinates, rather than new joiners, from a separate set of nests. In each of 34 nests that had not received joiners during our observations, we chose one of the lowest ranking subordinates and released it after removing the nest and the remaining nest residents, as in the joiner experiment, treatment 2.

Following the treatments, we searched for released joiners and subordinates in all nests in the sub-populations during daytime and evening censuses every 2–4 days. When a released individual was found on a new host nest with unmarked residents, we waited 2–3 days and then collected and marked the residents. We also resumed daytime censuses on all focal nests (including these new host nests) 2–3 days after treatment, and performed brood census as described above every 10–15 days. When the first worker(s) matured on a nest, we performed one final brood census, and then discontinued all censuses on that nest.

### (c) Video analysis

Each video was watched by one of seven people who recorded when nest residents left and returned to the nest, and all behavioural interactions. Observers were all trained by one person, who spot-checked for consistency. Behavioural interactions were ranked according to level of aggressiveness: antennation (given a value of 1), food sharing (2), and aggression (3; including all more aggressive encounters such as bite, chew and lunge). Two aggression values were calculated for each individual: the sum of values for all behaviours initiated by that individual, and the sum of values for all received behaviours during the full video recording.

Foraging returns brought back to the nest were ranked according to value in the following way: nothing visible (given a value of 0), nesting material (1), liquid food, as evidenced by trophallaxing (2), or a solid food ball (3). Foraging return values were calculated for each individual as the sum of values during the full video recording.

### (d) Genotyping and relatedness

Protocols were identical to those described previously [22]. Briefly, DNA was extracted from tarsus samples and samples were genotyped at nine microsatellite loci used previously in studies of the same population [3,22,44,46,47]. Each locus had between 6 and 51 different alleles in our samples (median in 2013 = 13; median in 2014 = 11). All loci were amplified in a single multiplex reaction using the Qiagen multiplex PCR kit (Qiagen, Venlo, The Netherlands).

Relatedness v. 5.0.8 software [48] was used to calculate relatedness between joiners and nest residents as in [22]. The Full Sibship Reconstruction procedure in Kingroup v. 2 software [44,49] was used to identify groups of sisters among the nests in each block (primary hypothesis: haplodiploid sisters; null hypothesis: haplodiploid cousins) [3]. We then counted the number of sisters each resident had in its own nest and in other nests. Only individuals with at least six out of nine loci scored successfully were used (median number of successful loci per sample = 9); 1996 out of 2011 wasps were successfully genotyped.

### (e) Statistics

All statistical analyses were performed using the statistical software R [50]. Whenever appropriate, non-parametric tests were used, and whenever the effect of more than one predictor was tested, GLMs (generalized linear models) or GLMMs (generalized linear mixed models) were used [51]. For count data we used Poisson error and tested for overdispersion: negative binomial error was used if models were overdispersed, and again we tested to ensure these models were no longer overdispersed before proceeding. For models with continuous data we used a Gaussian error structure and checked to ensure that residuals were homogeneous and normally distributed. Non-significant predictor variables (*p* > 0.05) were removed from full models in order to obtain more reliable *p*-values for the remaining predictors. When analysing data from video recordings, we incorporated nest ID and the ID of the person watching the video as random effects. When analysing aggression and foraging return values we used the glmmADMB package [52] to build GLMMs with negative binomial error. This package further allowed us to account for zero inflation in the aggression models.

## 3. Results

### (a) Joiners' alternative options

We permanently removed the first nest choices of 32 joiners and recovered 25 (78.1%) of them on their second nesting choices. Of these 25 second-choice joiners, 18 joined other established nests, three initiated new nests with other females, three joined nests of unknown ages, and only a single joiner definitely initiated a new nest alone.

Out of 21 second-choice joiners with known fates, six (28.6%) became the dominant breeder on their second-choice nest after joining or initiating it; the remaining 15 (71.4%) became subordinates. A first-choice joiner became the dominant breeder on 2 out of 14 control nests (14.3%) after joining. Thus, more joiners tended to become dominant through their second nest choice than through their first, although this difference was not significant (*χ*^2^ with Yates's correction = 0.33, d.f. = 1, *p* = 0.57).

**Figure 2.**
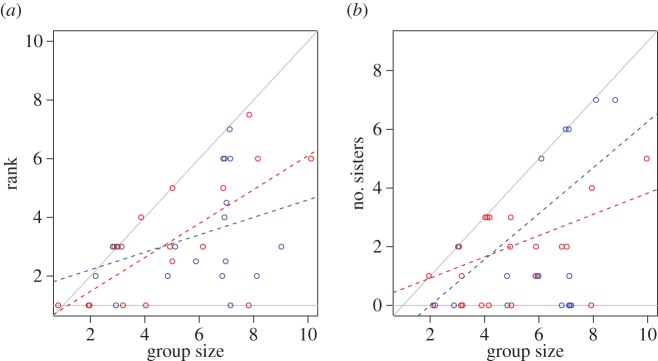
Joiners in their first and second nest choices. Rank obtained by joiners (*a*) and the number of sisters (*b*) in their first-choice (blue) and second-choice nests (red); points have been slightly jittered along the *x*-axis. Grey lines indicate the parameter space boundaries: if dots lie on the horizontal lines, a joiner had become the dominant breeder (i.e. rank 1) (*a*) or had zero sisters in its nest (*b*); if dots lie on the steep lines, joiners had become the lowest-ranked subordinates (*a*) or had only sisters in the group (*b*). Stippled lines indicate simple regression lines for first-choice (blue) and second-choice (red) joiners. Differences between first- and second-choice joiners were non-significant (rank: *p* = 0.80; number of sisters: *p* = 0.97).

Joiners' second choices were similar to their first choices in terms of other factors expected to affect fitness payoffs. Firstly, there was no difference between first- and second-choice joiners in the social rank they obtained after joining, correcting for group size (figure 2*a*; GLM, Poisson error; *y* = social rank after treatment; main effects: treatment: *z* = 0.26, *p* = 0.80, group size: *z* = 3.32, *p* < 0.001, interaction between treatment and group size: *z* = 0.95, *p* = 0.34, *n* *=* 36). Secondly, there was no difference between first- and second-choice joiners in terms of the joiners' genetic relatedness to the dominant in the group they joined (comparing first- and second-choice joiners after treatment: Mann–Whitney *U* test: *W* = 73, *p* = 0.64, *n* = 26; comparing second-choice joiners' first and second nest choices: Wilcoxon paired, *V* = 19, *p* = 0.95, *n* pairs = 8), or in the number of sisters they had in the group, correcting for group size (figure 2*b*; GLM, negative binomial error; *y* = number of sisters after treatment; main effects: first- versus second-choice joiners: *z* = 0.038, *p* = 0.97; group size: *z* = 3.31, *p* < 0.001; interaction between treatment and group size: *z* = −1.41, *p* = 0.16, *n* = 40; *y* = number of sisters of second-choice joiners; main effects: first versus second choice: *z* = −0.97, *p* = 0.33; group size: *z* = 3.68, *p* < 0.001; interaction between choice and group size: *z* = 0.09, *p* = 0.93, *n* = 22). Thirdly, there was no difference between first-choice joiners, second-choice joiners and established nest residents in whether they stayed in their groups until worker maturation or had disappeared by this stage (*χ*^2^ = 1.42, d.f. = 2, *p* = 0.49; first-choice joiners: 14 out of 20 (70.0%); second-choice joiners: 12 out of 23 (52.2%); established nest residents: 84 out of 141 (59.6%) stayed till worker maturation).

New nests of second-choice joiners were mainly located within a couple of metres of first nest choices (figure 1; median = 1.21 m, mean = 1.93 m, max. = 8.9 m). Seven out of 22 (31.8%) second-choice joiners chose the closest nest (of which five were established nests and two were newly initiated). It was relatively common for wasps to visit other nests in the population. We spotted 194 of the 1603 marked wasps in the population (12.1%) on at least two different nests. Wasps visited nests that were located up to 54.6 m away from their original nests, but more than 95% of them visited within a 5 m radius (median distance = 0.9 m; mean distance = 2.2 m).

### (b) Consequences of rejecting a joiner for established nest residents

**Figure 3.**
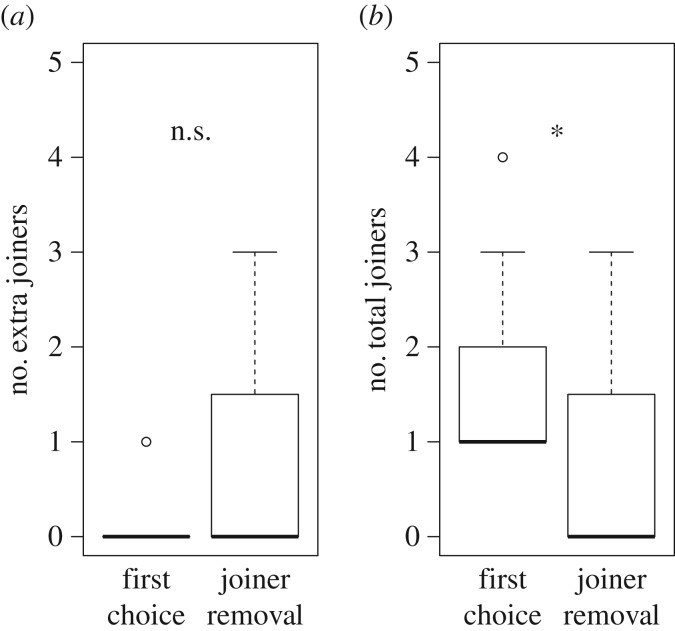
The number of joiners received in first-choice (control) nests and in joiner-removal nests. (*a*) The number of extra joiners that arrived after treatment (*p* = 0.37). (*b*) The total number of joiners received, including the treatment-joiners in control nests but excluding them in joiner-removal nests (*p* = 0.014).

We removed one or two joiners from each of 16 joiner-removal nests (21 joiners removed). After treatment, more joiner-removal nests received extra joiners (7 out of 16 nests: 43.8%) than did control nests where joiners were allowed to stay (2 out of 14 nests: 14.3%). However, the difference in number of extra joiners received in the two treatments was not significant (figure 3*a*; Mann–Whitney *U* test: *W* *=* 91.5, *p* = 0.37, *n* *=* 30), and the extra joiners received were not enough to replace those removed: control nests received significantly more joiners overall (including focal joiners) than joiner-removal nests did excluding removed focal joiners (figure 3*b*; Mann–Whitney *U* test: *W* *=* 169, *p* = 0.014, *n* *=* 30).

Original dominants were no more likely to lose their dominant breeding positions in control nests where joiners were allowed to stay than in joiner-removal nests. After treatment, the dominant lost her breeding position in 4 out of 13 (30.8%) control nests and in 4 out of 15 (26.7%) joiner-removal nests (*χ*^2^ with Yates's correction = 0, d.f. = 1, *p* = 1). Additionally, established nest residents were no more likely to leave their nests after a joining event in control compared to joiner-removal nests (Mann–Whitney *U* test: *W* *=* 116.5, *p* = 0.86, *n* nests = 30).

Nest success, measured as date of worker maturation and as brood development at worker maturation, was not affected by treatment or by a switch in the dominant breeder's identity. Only the number of nest residents and brood development at the time of treatment significantly affected brood development at worker maturation (both effects positive; GLM, *y* = date of worker emergence; main effects: treatment: *t* = −0.50, *p* = 0.78, dominance-usurpation: *t* = 1.51, *p* = 0.14, group size: *t* = −0.94, *p* = 0.35, brood value at joining: *t* = −1.69, *p* = 0.10; *y* = brood value at worker emergence; main effects: treatment: *t* = 0.45, *p* = 0.62, dominance-usurpation: *t* = 0.79, *p* = 0.94, group size: *t* = 3.59, *p* = 0.0012, brood value at joining: *t* = 4.64, *p* < 0.001; *n* nests = 55).

### (c) Behavioural interactions during joining events

New joiners did not spend more time foraging than established subordinates, correcting for rank (GLMM; Gaussian error; *y* = time spent off the nest; main effects: joiner or subordinate: *χ*^2^ = 1.63, *p* = 0.20, rank: *χ*^2^ = 95.57, *p* < 0.001). However, new joiners brought back a higher total value of foraging items than established subordinates, correcting for time spent foraging. In other words, forage value per time unit spent foraging was higher for recent joiners than for established subordinates. The amount of aggression that a joiner received also tended to be positively correlated with foraging return values, while relatedness between the joiner and the established nest residents had no effect on foraging returns (GLMM; negative binomial error; *y* = total foraging return value; main effects: joiner or subordinate: *z* = 2.73, *p* = 0.0063, aggression received: *z* = 1.71, *p* = 0.088, time spent off the nest: *z* = 1.75, *p* = 0.080, average relatedness between joiner and residents: *z* = −1.47, *p* = 0.14). Recent joiners that later became the dominant breeders on their nests worked less hard during video recordings than joiners that remained subordinate (*n* = 19; *y* = foraging return value per time unit; Wilcoxon's *W* = 8.5, *p* = 0.043).

Joiners neither received nor initiated more aggression than other nest residents, and average relatedness between joiner and residents did not affect aggression levels (GLMM; negative binomial error; *y* = aggression received; main effects: joiner or resident: *z* = 1.41, *p* = 0.16; number of days after joining: *z* = −0.20, *p* = 0.84; relatedness between joiner and residents: *z* = 0.33, *p* = 0.74; time spent on the nest: *z* = 3.52, *p* < 0.001; *y* = aggression initiated; main effects: joiner or resident: *z* = −1.11, *p* = 0.27, number of days after joining: *z* = −0.83, *p* = 0.41, relatedness between joiner and residents: *z* = 0.22, *p* = 0.82, time spent on the nest: initiated: *z* = 10.19, *p* < 0.001; *n* wasps = 142, *n* nests = 21).

### (d) Established subordinates' alternative options

Of 34 released subordinates, we relocated 18 (52.9%) on their second nesting choices: 10 joined other established nests; three initiated a new nest with each other; four joined nests of unknown ages; and only a single subordinate nested solitarily (taking over an abandoned nest). As with the second-choice joiners, the second nesting choices of released low-ranking subordinates were no different than their first choices with regard to inheritance rank obtained and presence of sisters (ranks: Wilcoxon paired, *V* = 63.5, *p* = 0.22, *n* = 15; presence of sisters: *χ*^2^ with Yates's correction = 0, d.f. = 1, *p* = 1). Released subordinates also mainly chose their new nests within a couple of metres (median = 1.30 m; mean = 1.42 m; max = 3.37 m).

## 4. Discussion

### (a) Joiners had high-payoff alternative options

We quantified the outside options available to cooperatively breeding paper wasps, *P. dominula*, and found that at the time of joining a nest, individuals had alternative options that offered potentially high fitness payoffs. After we experimentally removed their first nest choices, joiners' second nesting choices included a more than 1 : 4 chance of obtaining the dominant breeding position in a social group, which is the highest payoff possible in this species. This means that outside options offered much greater fitness payoffs than solitary nesting: at our field site, the payoff from solitary nesting is close to zero due to extremely high nest failure rates (more than 90% of solitary nests fail [3,40,53]). This result shows clearly that partner choice in *P. dominula* has the potential to affect payoff distributions in models predicting the amount of help provided by subordinates [7,21] or the amount of reproduction that dominants might have to concede to retain helpers [14,42,54]. Simply comparing payoffs from observed helping decisions with those from a default solitary breeding option, as is traditionally done, is likely to greatly overestimate the relative benefit of staying and helping in the current group. When high-quality outside options exist, dominants may accept a lower subordinate work effort than traditional models would predict. Hence, we demonstrate that multiplayer models, such as those offered by biological market theory, are more appropriate than traditional models for understanding levels of help in cooperative breeders such as *P. dominula* [7,9,21,22].

### (b) First and second nest choices offered similar payoffs

Joiners and established subordinates did not necessarily have to settle for inferior payoff options, compared with their first nesting choice, when forced to make a second choice, contrary to our predictions. This result suggests that individuals had more than one relatively high-quality option available in the market. We found that direct fitness returns associated with the chances of usurping or inheriting the breeding position, as well as indirect fitness returns from helping a related dominant, were no smaller in second choices than in first nesting choices. We predicted that joiners should evaluate their options and choose the one that offered the highest fitness payoff [10,21,53]. However, joiners may have insufficient information to make this choice: it is probably difficult for an individual to evaluate the exact chances of obtaining the breeding position in all nests in the market, prior to actually joining. Furthermore, there is little evidence that females can discriminate relatedness at the individual level in this species [39,44,55] and thereby preferentially join relatives to maximize inclusive fitness. Indeed, both joiners and subordinates sometimes chose to join nests without sisters despite having sisters in nearby nests. These results suggest that joiners chose one of several options available to them, each offering relatively high payoffs, indicating that the biological market is large [22].

### (c) Rejecting a joiner may be costly for dominant breeders

Experimentally removed joiners could not easily be replaced with new ones, suggesting that there is not an unlimited pool of potential joiners in the population (as was also found in *Polistes carolina* [20]). Additionally, nest success increased with the number of nest residents, substantiating previous findings that larger groups fare better [3,40] and that it is in the interest of dominants to accept joiners, particularly related ones, in order to increase group size. Taken together, these results suggest that, as we predicted, it may be costly for dominants to reject joiners. Supporting this result, we found that original dominants were no more likely to lose their dominant breeding positions when joiners were allowed to stay, compared with when joiners were removed. Hence, by accepting a joiner, an original dominant does not necessarily incur a cost in terms of an increased risk of nest usurpation, as she already faces a risk of losing her breeding position to one of her established subordinates. Allowing joiners to stay also did not generally make established subordinates more likely to leave.

We thus propose that a dominant cannot afford to be too ‘choosy’ when presented with potential joiners: it is in her interest to increase group size [3], so long as the risk of the joiner usurping dominance is not too high. This potentially makes joiners the ‘choosers’ in the market [10], so that dominants are effectively competing with each other to attract a limited supply of joiners. Dominants may therefore be prepared to accept a reduced workload from subordinates in order to retain them when competition for help increases in the population [22].

### (d) Joiners may pay for group membership

Rather than using aggression, joiners may have used appeasement in the form of ‘pay to stay’ in order to become accepted in their new nests. Within the first few days of joining, we found that joiners provided higher-value forage than other subordinates on their nests, perhaps to ‘pay’ for acceptance by the group. Furthermore, joiners were not involved in a disproportionate number of aggressive interactions, contrary to what would be expected if they were ‘forcing’ their acceptance as new residents. These results render support for the hypothesis that subordinates trade helping behaviour in return for group membership [15]: when first arriving at a nest, a joiner may need to prove her worth and convey she does not represent a high risk of usurping the breeding position [15,32].

An alternative to the general idea that a joiner works in exchange for group membership is that she works simply because any investment in the nest would directly benefit her if she later took over the dominant breeding position herself (‘group augmentation’ [56]). However, our findings do not support the hypothesis that subordinates are maximizing only group augmentation benefits, because joiners that later became dominants worked *less* hard than those that remained subordinate. This is consistent with previous findings that group members higher up the hierarchy, and therefore more likely to inherit the dominant position, in fact work less hard than lower-ranked subordinates [45].

### (e) Sampling costs and prospecting

Given that experimentally presented foreign conspecifics are normally attacked by nest residents [44], the lack of aggression towards new joiners suggests that residents may have already been familiar with joiners, perhaps through previous visits by joiners to establish familiarity via ‘prospecting behaviour’ [11,57]. Individuals in cooperative species may benefit from maintaining a social network outside their current groups by visiting and familiarizing themselves with members of other groups. This prospecting behaviour can provide them with information about whether between-group dispersal would be beneficial, and can maximize their chances of being accepted in the new group, should they be expelled from their current group, choose to leave or if their nest fails [11,57,58]. Indeed, nests fail at high rates in *P. dominula* [3,40], and prospecting behaviour may be common: we spotted 12.1% of marked wasps on at least two different nests. This number is similar to previous studies of the same population (approx. 16% in [40] and approx. 14% in [22]).

However, prospecting behaviour is likely to be costly [59] as visiting other groups requires time and energy that could otherwise be spent foraging. These costs, called sampling costs or searching costs in biological market terms [9,10], are likely to limit the number of groups a subordinate wasp can maintain in its social network. Second choice joiners mainly chose options that were nearby. This may partly be because nests containing genetic relatives tend to be nearby, but greater costs of prospecting further afield could also contribute. In a scenario where sampling costs are very high, for example in a very low-density population where maintaining peaceful relationships with distant neighbouring groups would pose a high risk of predation or great energetic expenditure, market forces could fail to operate, as there might effectively be no outside options available [10]. Hence, quantifying sampling costs and documenting the actual social networks that individuals gain through prospecting is an important avenue for further studies in this system.

## 5. Conclusion

### (a) Invoking biological market models to include outside options

Our key finding is that in a cooperatively breeding paper wasp, *P. dominula*, both new joiners and established subordinates have alternative nesting options that offer fitness payoffs comparable with their first nest choices and that are higher than the payoff through solitary nesting. The existence of multiple options with similar payoffs has important implications for the conditions that subordinates should accept in their groups; or in biological market terms, the deal settled on between trading partners. For example, high-quality outside options will affect the trade value of helping behaviour and therefore influence how much help subordinates are prepared to provide with rearing the dominant's offspring [22]. Outside options may also determine whether subordinates should demand a share of the reproduction in return for their services in species where reproductive concessions are likely to occur [23,60,61]. Hence, our findings clearly suggest that biological market models are indeed necessary for understanding helping behaviour in *P. dominula*. This result is relevant for a wide range of cooperatively breeding species where successful dispersal among groups occurs (for example cichlids [11], carrion crows [62], dwarf mongooses [60], baboons [63]). Unlike traditional models, which assume that a subordinate's only alternative is solitary breeding, market models allow for partner choice, partner switching and competition for partners [9,10]. To conclude, traditional cooperative theory and reproductive skew models are therefore likely to overestimate subordinates' propensity to stay and help in their group, overestimate the level of help that they provide, and perhaps underestimate the level of reproductive concession the dominant should offer her helpers. Future studies should identify and quantify the alternative options available, and include these in models predicting the rate of exchange of cooperative behaviours within groups of cooperatively breeding species.

## Data accessibility

All data are made available online via Dryad: http://dx.doi.org/10.5061/dryad.58c8v [64].

## References

[1] Cockburn A 1998 Evolution of helping behavior in cooperatively breeding birds. Annu. Rev. Ecol. Syst. 29, 141–177. (10.1146/annurev.ecolsys.29.1.141)

[2] Clutton-Brock T 2002 Behavioral ecology—breeding together: kin selection and mutualism in cooperative vertebrates. Science 296, 69–72. (10.1126/science.296.5565.69) 11935014

[3] Leadbeater E, Carruthers JM, Green JP, Rosser NS, Field J 2011 Nest inheritance is the missing source of direct fitness in a primitively eusocial insect. Science 333, 874–876. (10.1126/science.1205140) 21836014

[4] Kokko H, Johnstone RA 1999 Social queuing in animal societies: a dynamic model of reproductive skew. Proc. R. Soc. Lond. B 266, 571–578. (10.1098/rspb.1999.0674)

[5] Hamilton WD 1964 Genetical evolution of social behaviour. I. J. Theor. Biol. 7, 1 (10.1016/0022-5193(64)90038-4) 5875341

[6] Griffin AS, West SA 2003 Kin discrimination and the benefit of helping in cooperatively breeding vertebrates. Science 302, 634–636. (10.1126/science.1089402) 14576431

[7] Bergmuller R, Johnstone RA, Russell AF, Bshary R 2007 Integrating cooperative breeding into theoretical concepts of cooperation. Behav. Processes 76, 61–72. (10.1016/j.beproc.2007.07.001) 17703898

[8] Field J, Cant MA 2007 Direct fitness, reciprocity and helping: a perspective from primitively eusocial wasps. Behav. Processes 76, 160–162. (10.1016/j.beproc.2007.01.019) 17719185

[9] Noë R, Hammerstein P 1995 Biological markets. Trends Ecol. Evol. 10, 336–339. (10.1016/S0169-5347(00)89123-5) 21237061

[10] Noë R, Hammerstein P 1994 Biological markets—supply-and-demand determine the effect of partner choice in cooperation, mutualism and mating. Behav. Ecol. Sociobiol. 35, 1–11. (10.1007/BF00167053)

[11] Bergmuller R, Heg D, Peer K, Taborsky M 2005 Extended safe havens and between-group dispersal of helpers in a cooperatively breeding cichlid. Behaviour 142, 1643–1667. (10.1163/156853905774831800)

[12] Schino G, Aureli F 2016 Reciprocity in group-living animals: partner control versus partner choice. Biol. Rev. 92, 665–672. (10.1111/brv.12248) 26733357

[13] Vehrencamp SL 1983 A model for the evolution of despotic versus egalitarian societies. Anim. Behav. 31, 667–682. (10.1016/S0003-3472(83)80222-X)

[14] Keller L, Reeve HK 1994 Partitioning of reproduction in animal societies. Trends Ecol. Evol. 9, 98–102. (10.1016/0169-5347(94)90204-6) 21236786

[15] Kokko H, Johnstone RA, Wright J 2002 The evolution of parental and alloparental effort in cooperatively breeding groups: when should helpers pay to stay? Behav. Ecol. 13, 291–300. (10.1093/beheco/13.3.291)

[16] Reeve HK, Ratnieks FLW 1993 Queen conflict in polygynous societies: mutual tolerance and reproductive skew. In Queen number and sociality in insects (ed. Keller L), pp. 45–85 Oxford, UK: Oxford University Press.

[17] Nonacs P, Liebert AE, Starks PT 2006 Transactional skew and assured fitness return models fail to predict patterns of cooperation in wasps. Am. Nat. 167, 467–480. (10.1086/501168) 16670991

[18] Reeve HK, Emlen ST, Keller L 1998 Reproductive sharing in animal societies: reproductive incentives or incomplete control by dominant breeders? Behav. Ecol. 9, 267–278. (10.1093/beheco/9.3.267)

[19] Jennions MD, Macdonald DW 1994 Cooperative breeding in mammals. Trends Ecol. Evol. 9, 89–93. (10.1016/0169-5347(94)90202-X) 21236784

[20] Seppä P, Queller DC, Strassmann JE 2012 Why wasp foundresses change nests: relatedness, dominance, and nest quality. PLoS ONE 7, e45386 (10.1371/journal.pone.0045386) 23049791 PMC3458021

[21] Noë R, van Schaik CP, van Hooff JARAM 1991 The market effect—an explanation for pay-off asymmetries among collaborating animals. Ethology 87, 97–118. (10.1111/j.1439-0310.1991.tb01192.x)

[22] Grinsted L, Field J 2016 Market forces influence helping behaviour in cooperatively breeding paper wasps. Nat. Commun. 8, 13750 (10.1038/ncomms13750) PMC5286204 28117836

[23] Reeve HK 1998 Game theory, sibling rivalry, and parent–offspring conflict In Game theory and animal behavior (eds Dugatkin LA, Reeve HK), pp. 118–145. Oxford, UK: Oxford University Press.

[24] Johnstone RA, Bshary R 2008 Mutualism, market effects and partner control. J. Evol. Biol. 21, 879–888. (10.1111/j.1420-9101.2008.01505.x) 18312320

[25] Bshary R, Grutter AS 2002 Experimental evidence that partner choice is a driving force in the payoff distribution among cooperators or mutualists: the cleaner fish case. Ecol. Lett. 5, 130–136. (10.1046/j.1461-0248.2002.00295.x)

[26] Bull JJ, Rice WR 1991 Distinguishing mechanisms for the evolution of cooperation. J. Theor. Biol. 149, 63–74. (10.1016/S0022-5193(05)80072-4) 1881147

[27] Dugatkin LA, Mesterton-Gibbons M, Houston AI 1992 Beyond the prisoner's dilemma—toward models to discriminate among mechanisms of cooperation in nature. Trends Ecol. Evol. 7, 202–205. (10.1016/0169-5347(92)90074-L) 21236008

[28] Brembs B 1996 Chaos, cheating and cooperation: potential solutions to the prisoner's dilemma. Oikos 76, 14–24. (10.2307/3545744)

[29] Hamilton IM, Taborsky M 2005 Unrelated helpers will not fully compensate for costs imposed on breeders when they pay to stay. Proc. R. Soc. B 272, 445–454. (10.1098/rspb.2004.2961) PMC1634984 15734700

[30] Gaston AJ 1978 Evolution of group territorial behavior and cooperative breeding. Am. Nat. 112, 1091–1100. (10.1086/283348)

[31] Kutsukake N, Clutton-Brock TH 2010 Grooming and the value of social relationships in cooperatively breeding meerkats. Anim. Behav. 79, 271–279. (10.1016/j.anbehav.2009.10.014)

[32] Bergmuller R, Taborsky M 2005 Experimental manipulation of helping in a cooperative breeder: helpers ‘pay to stay’ by pre-emptive appeasement. Anim. Behav. 69, 19–28. (10.1016/j.anbehav.2004.05.009)

[33] Lottker P, Huck M, Zinner DP, Heymann EW 2007 Grooming relationships between breeding females and adult group members in cooperatively breeding moustached tamarins (*Saguinus mystax*). Am. J. Primatol. 69, 1159–1172. (10.1002/ajp.20411) 17330867

[34] Lazaro-Perea C, De Fatima M, Snowdon CT 2004 Grooming as a reward? Social function of grooming between females in cooperatively breeding marmosets. Anim. Behav. 67, 627–636. (10.1016/j.anbehav.2003.06.004) 17237884 PMC1761567

[35] Bergmuller R, Heg D, Taborsky M 2005 Helpers in a cooperatively breeding cichlid stay and pay or disperse and breed, depending on ecological constraints. Proc. R. Soc. B 272, 325–331. (10.1098/rspb.2004.2960) PMC1634971 15705559

[36] Fischer S, Zottl M, Groenewoud F, Taborsky B 2014 Group-size-dependent punishment of idle subordinates in a cooperative breeder where helpers pay to stay. Proc. R. Soc. B 281, 20140184 (10.1098/rspb.2014.0184) PMC4100499 24990673

[37] Mulder RA, Langmore NE 1993 Dominant males punish helpers for temporary defection in superb fairy-wrens. Anim. Behav. 45, 830–833. (10.1006/anbe.1993.1100)

[38] Dunn PO, Cockburn A, Mulder RA 1995 Fairy-wren helpers often care for young to which they are unrelated. Proc. R. Soc. Lond. B 259, 339–343. (10.1098/rspb.1995.0050)

[39] Field J, Leadbeater E 2016 Cooperation between non-relatives in a primitively eusocial paper wasp, *Polistes dominula*. Phil. Trans. R. Soc. B 371, 20150093 (10.1098/rstb.2015.0093) 26729932 PMC4760194

[40] Zanette LRS, Field J 2011 Founders versus joiners: group formation in the paper wasp *Polistes dominulus*. Anim. Behav. 82, 699–705. (10.1016/j.anbehav.2011.06.025)

[41] Reeve HK 1991 Polistes. In The social biology of wasps (eds Ross KG, Matthews RW), pp. 99–148. Ithaca, NY: Cornell University Press.

[42] Liebert AE, Starks PT 2006 Taming of the skew: transactional models fail to predict reproductive partitioning in the paper wasp *Polistes dominulus*. Anim. Behav. 71, 913–923. (10.1016/j.anbehav.2005.09.005)

[43] Queller DC et al. 2000 Unrelated helpers in a social insect. Nature 405, 784–787. (10.1038/35015552) 10866197

[44] Leadbeater E, Carruthers JM, Green JP, van Heusden J, Field J 2010 Unrelated helpers in a primitively eusocial wasp: is helping tailored towards direct fitness? PLoS ONE 5, 1–7. (10.1371/journal.pone.0011997) PMC2917371 20700463

[45] Cant MA, Field J 2001 Helping effort and future fitness in cooperative animal societies. Proc. R. Soc. Lond. B 268, 1959–1964. (10.1098/rspb.2001.1754) PMC1088835 11564355

[46] Henshaw MT 2000 Microsatellite loci for the social wasp *Polistes dominulus* and their application in other polistine wasps. Mol. Ecol. 9, 2155–2157. (10.1046/j.1365-294X.2000.01053.x) 11123629

[47] Strassmann JE, Barefield K, Solis CR, Hughes CR, Queller DC 1997 Trinucleotide microsatellite loci for a social wasp, Polistes. Mol. Ecol. 6, 97–100. (10.1046/j.1365-294X.1997.00158.x) 9004523

[48] Queller DC, Goodnight KF 1989 Estimating relatedness using genetic-markers. Evolution 43, 258–275. (10.2307/2409206) 28568555

[49] Konovalov DA, Manning C, Henshaw MT 2004 KINGROUP: a program for pedigree relationship reconstruction and kin group assignments using genetic markers. Mol. Ecol. Notes 4, 779–782. (10.1111/j.1471-8286.2004.00796.x)

[50] R Core Team. 2014 R: a language and environment for statistical computing. Vienna, Austria: R Foundation for Statistical Computing See http://www.R-project.org/.

[51] Bates D, Maechler M, Bolker B 2011 lme4: Linear mixed-effects models using S4 classes. See http://CRAN.R-project.org/package=lme4.

[52] Fournier DA et al. 2012 AD Model Builder: using automatic differentiation for statistical inference of highly parameterized complex nonlinear models. Optim. Methods Softw. 27, 233–249. (10.1080/10556788.2011.597854)

[53] Nonacs P, Reeve HK 1995 The ecology of cooperation in wasps—causes and consequences of alternative reproductive decisions. Ecology 76, 953–967. (10.2307/1939359)

[54] Field J, Cant MA 2009 Reproductive skew in primitively eusocial wasps: how useful are current models? In *Reproductive skew in vertebrates: proximate and ultimate causes* (eds R Hager, CB Jones), pp. 305–334. Cambridge, UK: Cambridge University Press.

[55] Leadbeater E, Dapporto L, Turillazzi S, Field J 2014 Available kin recognition cues may explain why wasp behavior reflects relatedness to nest mates. Behav. Ecol. 25, 344–351. (10.1093/beheco/art113)

[56] Kokko H, Johnstone RA, Clutton-Brock TH 2001 The evolution of cooperative breeding through group augmentation. Proc. R. Soc Lond. B 268, 187–196. (10.1098/rspb.2000.1349) PMC1088590 11209890

[57] Jungwirth A, Walker J, Taborsky M 2015 Prospecting precedes dispersal and increases survival chances in cooperatively breeding cichlids. Anim. Behav. 106, 107–114. (10.1016/j.anbehav.2015.05.005)

[58] Jordan LA, Wong MYL, Balshine SS 2010 The effects of familiarity and social hierarchy on group membership decisions in a social fish. Biol. Lett. 6, 301–303. (10.1098/rsbl.2009.0732) 20007168 PMC2880034

[59] Young AJ, Carlson AA, Clutton-Brock T 2005 Trade-offs between extraterritorial prospecting and helping in a cooperative mammal. Anim. Behav. 70, 829–837. (10.1016/j.anbehav.2005.01.019)

[60] Creel SR, Waser PM 1991 Failures of reproductive suppression in dwarf mongooses (*Helogale parvula*): accident or adaptation? Behav. Ecol. 2, 7–15. (10.1093/beheco/2.1.7)

[61] Clutton-Brock TH 1998 Reproductive skew, concessions and limited control. Trends Ecol. Evol. 13, 288–292. (10.1016/S0169-5347(98)01402-5) 21238306

[62] Baglione V, Marcos JM, Canestrari D, Ekman J 2002 Direct fitness benefits of group living in a complex cooperative society of carrion crows, *Corvus corone corone*. Anim. Behav. 64, 887–893. (10.1006/anbe.2002.2007)

[63] Alberts SC, Altmann J 1995 Balancing costs and opportunities—dispersal in male baboons. Am. Nat. 145, 279–306. (10.1086/285740)

[64] Grinsted L, Field J 2017 Data from: Biological markets in cooperative breeders: quantifying outside options. Data Dryad Repository. (10.5061/dryad.58c8v) PMC5474085 28615504

